# Association of metabolic syndrome and electrocardiographic markers of subclinical cardiovascular disease

**DOI:** 10.1186/s13098-017-0238-9

**Published:** 2017-05-22

**Authors:** Theodora W. Elffers, Renée de Mutsert, Hildo J. Lamb, Arie C. Maan, Peter W. Macfarlane, Ko Willems van Dijk, Frits R. Rosendaal, J. Wouter Jukema, Stella Trompet

**Affiliations:** 1 Department of Clinical Epidemiology, Leiden University Medical Center, PO Box 9600, 2300RC Leiden, The Netherlands; 2 Department of Cardiology, Leiden University Medical Center, 2300RC Leiden, The Netherlands; 3 Department of Radiology, Leiden University Medical Center, 2300RC Leiden, The Netherlands; 4 Institute of Health and Wellbeing, University of Glasgow, Glasgow, UK; 5 Department of Human Genetics, Leiden University Medical Center, 2300RC Leiden, The Netherlands; 6 Department of Medicine, Division Endocrinology, Leiden University Medical Center, 2300RC Leiden, The Netherlands; 7 Department of Gerontology and Geriatrics, Leiden University Medical Center, 2300RC Leiden, The Netherlands

**Keywords:** Metabolic syndrome, Obesity, Cardiovascular disease

## Abstract

**Background:**

The metabolic syndrome (MetS) and its components are well-established risk factors for cardiovascular diseases (CVD). It is inconclusive whether MetS and MetS score are associated with electrocardiographic markers of subclinical CVD, therefore we investigated this in a population without pre-existing CVD.

**Methods:**

We performed a cross-sectional analysis in the Netherlands Epidemiology of Obesity study, a population-based cohort including 6671 participants aged 45–65. We excluded participants with pre-existing CVD (n = 499) or missing MetS components (n = 58). MetS was defined based on a modified definition of Adult Treatment Panel III. Subclinical CVD parameters were determined with 12-lead ECGs. MetS score was defined as number of abnormal MetS components and obesity as Body Mass Index (BMI) ≥30 kg/m^2^. We performed weighted adjusted linear regression analyses.

**Results:**

Our study population (n = 6114) had a mean (SD) BMI of 26.3 (4.4) kg/m^2^ and MetS was present in 24% of participants. All ECG parameters differed between participants with and without MetS. Per additional MetS component, heart rate was 0.17 SD (95% CI 0.15, 0.19) higher, P wave duration, QRS complex duration and corrected QT interval were longer [0.07 SD (0.05, 0.10), 0.04 SD (0.01, 0.06) and 0.05 SD (0.02, 0.08) respectively], P wave axis, T wave axis and QRS axis were lower [−0.10 SD (−0.12, −0.07), −0.07 SD (−0.10, −0.05) and −0.19 SD (−0.22, −0.16)] and percentage small Q-waves also increased per additional MetS component. Associations were stronger in non-obese than obese participants. In joint modelling of all MetS components, increased waist circumference showed strongest associations with ECG parameters.

**Conclusions:**

Metabolic syndrome score and its individual components, in particular abdominal obesity, are associated with ECG markers of subclinical CVD, showing the importance of limiting the amount of MetS components in both obese and non-obese persons.

**Electronic supplementary material:**

The online version of this article (doi:10.1186/s13098-017-0238-9) contains supplementary material, which is available to authorized users.

## Background

Cardiovascular disease (CVD) is the number one cause of death worldwide [1]. Metabolic syndrome (MetS) is a combination of cardiovascular risk factors such as obesity, hyperglycemia, dyslipidemia and hypertension, and has been associated with increased risk of CVD. A recent meta-analysis showed that individuals with MetS had a twofold increased risk of CVD and a 1.5-fold increased risk of all-cause mortality [2]. In a study including 4122 participants with a mean follow-up of 8.5 years, the risk of coronary heart disease increased with increasing number of MetS components [3]. In addition, there are also studies showing that separate components are more important for the risk of different outcomes than the combination of components in MetS [4, 5]. Obesity is a key component in MetS, becoming more relevant because of its increasing prevalence. However, there are also people who are metabolically unhealthy, but non-obese. Also, in these individuals there is a higher prevalence of diabetes and CVD [6]. Therefore it is important to understand the relation between MetS and its components and subclinical CVD in both non-obese and obese subpopulations.

Some previous studies investigated the association between MetS and subclinical CVD. Presence of MetS was associated with increased arterial stiffness, higher resting heart rate, prolonged QRS and prolonged QT interval and an abnormal T-wave axis [7, 8]. However, associations of MetS with subtle changes in electrocardiographic markers, indicative of subclinical CVD, are not fully elucidated. Few studies investigated these associations in both non-obese and obese subpopulations. Knowledge on these associations may give more insight in possible population consequences of subtle ECG changes. Therefore, our objectives were to investigate the associations of MetS with subclinical CVD, of the number of abnormal MetS components with subclinical CVD and to investigate whether these associations differ between obese and non-obese individuals without pre-existing CVD within the Netherlands Epidemiology of Obesity (NEO) study. Furthermore, we investigated contributions of separate MetS components to subclinical CVD.

## Methods

### Study design and population

The NEO study is a population-based, prospective cohort study comprising 6671 individuals, included between 2008 and 2012 and aged between 45 and 65 years [9]. Participants with BMI of 27 kg/m^2^ or higher were oversampled and also all inhabitants aged between 45 and 65 years from one municipality (Leiderdorp) were invited irrespective of their BMI, allowing for a reference distribution of BMI. Participants were invited to a baseline visit at the NEO study centre of the Leiden University Medical Center (LUMC) after an overnight fast. During this visit all participants underwent an extensive physical examination, including blood sampling and ECG. Participants completed a questionnaire with demographic, lifestyle, and clinical information. We excluded participants with missing values of waist circumference, glucose, triglycerides, HDL-cholesterol or blood pressure. Furthermore, participants with pre-existing CVD, defined as myocardial infarction, angina, congestive heart failure, stroke, or peripheral vascular disease, were excluded. The Medical Ethical Committee of the LUMC approved the design of the study. All participants gave informed consent.

### Data collection

Ethnicity was reported by the participants in eight categories and grouped into white and other. Level of education was reported in 10 categories according to the Dutch education system and grouped as low (none, primary school of lower vocational education) versus high. Tobacco smoking was categorized into current, former, or never smoker. Alcohol consumption was reported using a food frequency questionnaire and calculated into grams/day. Physical activity was reported using the Short Questionnaire to Assess Health-enhancing physical activity (SQUASH) questionnaire [10]. Energy expended during physical activity was calculated in leisure time in hours per week of metabolic equivalents (MET-h/week). Participants were asked to bring the medication they were currently using to the study visit and to report their medical history of diabetes or CVD. Height and weight were measured without shoes and 1 kg was subtracted from the weight to correct for clothing. BMI was calculated by dividing weight in kilograms by height in meters squared. Waist circumference was measured with a horizontally placed flexible tape in the middle of the distance between the lowest rib and the iliac crest. Brachial blood pressure was measured in seated position on the right arm using a validated automatic oscillometric device (OMRON, Model M10-IT, Omron Health Care Inc, IL, USA). Blood pressure was measured three times with 5 min rest between consecutive measurements. Mean systolic and diastolic blood pressure were calculated. Blood plasma was sampled after an overnight fast of 10 h. Fasting glucose, triglyceride and high-density lipoprotein concentrations were measured with the enzymatic colorimetric method (Roche Modular Analytics P800, Roche Diagnostics Mannheim, Germany).

A 12-lead ECG was obtained using a Mortara Eli-350 electrocardiograph (Mortara Instrument Inc., Best, the Netherlands) after a resting period of at least 10 min. ECGs were analysed using the automatic MATLAB-based (The MathWorks, Natick, MA) program BEATS and the semiautomatic program LEADS [11, 12]. ECGs were also provided to the University of Glascow ECG core lab where Minnesota Codes were determined [13–15]. In order to assess subtle changes in ECG parameters, that could indicate subclinical CVD in a population without known overt cardiovascular diseases, heart rate, P wave duration, QRS complex duration, PR interval, corrected QT interval (corrected according to the Bazett formula), P-, T- and QRS axis were determined. Furthermore, small Q-waves were assessed using the Minnesota Coding System, which is a system to objectively describe electrocardiographic findings. We defined small Q-waves as Minnesota Codes 1.2.x or 1.3.x.

These ECG parameters are known to be of prognostic importance for CVD development [16–19].

### Metabolic syndrome definition

The definition of MetS as proposed by the National Cholesterol Education Program Adult Treatment Panel III was used, with minor modifications as stated in the American Heart Association and the National Heart, Lung, and Blood Institute statement [20]. MetS is defined as the presence in an individual of at least three out of the five following criteria: (1) increased waist circumference (>102 cm for men, >88 cm for women); (2) raised serum triglyceride levels (1.7 mmol/L) or on drug treatment to reduce triglyceride concentrations; (3) reduced serum HDL-cholesterol levels (1.03 mmol/L for men, 1.3 mmol/L for women) or on drug treatment to elevate HDL-cholesterol; (4) raised blood pressure (≥130 mmHg systolic/≥85 mmHg diastolic) or on antihypertensive drug treatment; (5) raised fasting plasma glucose (5.56 mmol/L) or on drug treatment to lower glucose concentrations. Obesity is defined as BMI ≥30 kg/m^2^ and MetS score as total number of MetS components present in an individual.

### Statistical analysis

To correctly represent associations in the general population, adjustments for the oversampling of participants with BMI of 27 kg/m^2^ or higher were made by weighting individuals towards the BMI distribution of participants from the Leiderdorp municipality whose BMI distribution was similar to that of the general Dutch population [21, 22].

Baseline characteristics were summarized as mean (SD) or percentage and stratified by the presence of MetS. We examined the associations between MetS and the ECG parameters heart rate (bpm), P wave duration (ms), QRS complex duration (ms), PR interval (ms), corrected QT interval (ms), P- (°), T- (°), QRS axis (°) and percentage of small Q-waves using linear regression analysis.

We calculated Z-scores and standardized the ECG parameters to a mean of zero with a standard deviation of one, in order to express the associations in SDs and compare them with each other. Next, we performed linear regression analyses with MetS score (0–5) as the independent and standardized ECG parameters as the outcome variables. To investigate differences in associations between obese and non-obese persons, we tested for interaction with obesity by including product terms of obesity and MetS in all models. Finally, to investigate the separate contributions of the MetS components in relation to the ECG parameters, we included all MetS components (dichotomous) in one joint model.

Crude associations were adjusted for age, sex, ethnicity, smoking, alcohol intake, education level, physical activity, and statin use. Data were analysed using STATA version 14.

## Results

Of the 6671 participants included in the NEO study, we consecutively excluded participants with a history of CVD (n = 499), missing data on fasting plasma glucose or glucose lowering therapy (n = 45), blood pressure or use of antihypertensive therapy (n = 7), waist circumference (n = 4) and serum triglycerides or use of medication to reduce triglyceride concentrations (n = 2). We ultimately included 6114 participants. Baseline characteristics are shown in Table 1. Of the participants, 24% met MetS criteria. Participants with MetS were more often men, current or former smoker and had a lower educational level. By definition, all other cardiovascular risk factors were more often present in the participants with MetS.

**Table 1:** Baseline characteristics of NEO study population without pre-existing CVD

	Metabolic syndrome	
	No (76%)	Yes (24%)
Age, years	55.3 (5.2)	56.5 (7.9)
Sex, men, %	39.9	52.6
Ethnicity, white, %	95	96
BMI, kg/m^2^	25.0 (3.0)	30.1 (6.4)
Smoking, %		
Never	40.8	33.5
Former	44.3	48.0
Current	14.9	18.5
Alcohol intake, g/dag	9.7 (3.2–20.9)	10.4 (2.0–24.6)
Physical activity, MET-hour/week	30.9 (16.9–51.0)	25.8 (11.8–45.2)
Education level, low, (%)^a^	16.0	28.0
Fasting plasma glucose, mg/dL	93.7 (88.3–98.9)	105.7 (98.9–114.2)
Use of glucose lowering therapy, %	0.6	7.6
Systolic blood pressure (mmHg)	127.6 (14.1)	137.6 (22.6)
Diastolic blood pressure (mmHg)	81.7 (8.5)	87.9 (13.4)
Use of antihypertensive therapy, %	14.5	40.3
Waist circumference (cm)		
Men	94.4 (7.7)	106.9 (13.9)
Women	83.6 (9.0)	101.1 (15.4)
Triglycerides, mg/dL	78.8 (59.3–106.3)	160.3 (111.6–208.1)
HDL, mg/dL		
Men	55.8 (11.0)	43.3 (15.4)
Women	71.5 (13.9)	53.4 (18.8)
Use of lipid lowering therapy, %	4.8	18.8

Data are presented as mean (SD), median (IQR) or percentage. Results were based on analyses weighted towards the BMI distribution of the general population

*BMI* Body Mass Index, *MET* metabolic equivalent of task

^a^Lower education: none, primary school, lower vocational education

Crude and adjusted weighted linear regression analyses were performed to compare ECG parameters between participants with and without MetS. Participants with MetS had higher values of heart rate, P wave duration, QRS complex duration, PR interval and corrected QT interval, and lower values of P, T and QRS axis, i.e. more superiorly oriented axes. Furthermore, small Q-waves were more often present in participants with MetS (Additional file 1: Table S1). Table 2 shows this analysis for the non-obese and obese participants separately. In the non-obese population, heart rate (difference: 5.0 bpm; 95% CI 3.9, 6.2), P wave duration (2.3 ms; 0.9, 3.6), P axis (−4.3°; −6.8, −1.9), T axis (−2.8°; −5.5, −0.1), QRS axis (−13.3°; −16.6, −10.1) and small Q-waves (2.8%; 0.2, 5.4) differed between participants with and without MetS and in the obese population, there were differences in heart rate (3.1 bpm; 2.2, 4.0), corrected QT interval (2.2 ms; 0.2, 4.2) and QRS axis (−2.9°; −5.3, −0.5).

**Table 2:** Differences in baseline ECG parameters between non-obese and obese participants with and without metabolic syndrome

	Non-obese (84%)			Obese (16%)			
ECG parameter	No MetS (83%)	MetS (17%)	Diff (95% CI)	No MetS (n = 36%)	MetS (64%)	Diff (95% CI)	*p* value int
Heart rate (bpm)	62.4 (0.2)	67.7 (0.5)	5.0 (3.9, 6.2)	65.8 (0.3)	68.8 (0.3)	3.1 (2.2, 4.0)	0.006
P duration (ms)	110.9 (0.4)	114.3 (0.6)	2.3 (0.9, 3.6)	115.2 (0.5)	115.8 (0.4)	−0.6 (−1.8, 0.6)	0.002
QRS duration (ms)	92.3 (0.3)	94.5 (0.5)	0.8 (−0.4, 2.0)	92.9 (0.4)	95.0 (0.3)	0.7 (−0.3, 1.7)	0.985
PR interval (ms)	162.1 (0.6)	166.3 (1.0)	2.4 (−0.1, 4.8)	163.9 (0.8)	164.8 (0.6)	−1.1 (−3.0, 0.9)	0.029
QTc interval (ms)	413.7 (0.6)	414.7 (1.0)	1.2 (−1.0, 3.4)	416.1 (0.9)	418.2 (0.6)	2.2 (0.2, 4.2)	0.373
P axis (°)	49.0 (0.7)	44.2 (1.0)	−4.3 (−6.8, −1.9)	40.4 (0.8)	40.6 (0.6)	−0.2 (−2.3, 1.9)	0.001
T axis (°)	39.4 (0.6)	36.2 (1.1)	−2.8 (−5.5, −0.1)	32.3 (0.8)	34.4 (0.7)	2.0 (−0.2, 4.2)	0.001
QRS axis (°)	39.8 (0.8)	23.0 (1.4)	−13.3 (−16.6, −10.1)	23.2 (1.0)	18.4 (0.8)	−2.9 (−5.3, −0.5)	<0.001
Small Q-wave (%)	5.2	9.1	2.8 (0.2, 5.4)	8.1	9.3	0.7 (−1.7, 3.0)	0.121

Data are presented as mean (se) or percentage

Results were based on weighted linear regression analysis adjusted for age, sex, ethnicity, smoking, alcohol intake, education level, physical activity and statin use

*MetS* metabolic syndrome

**Fig. 1:**
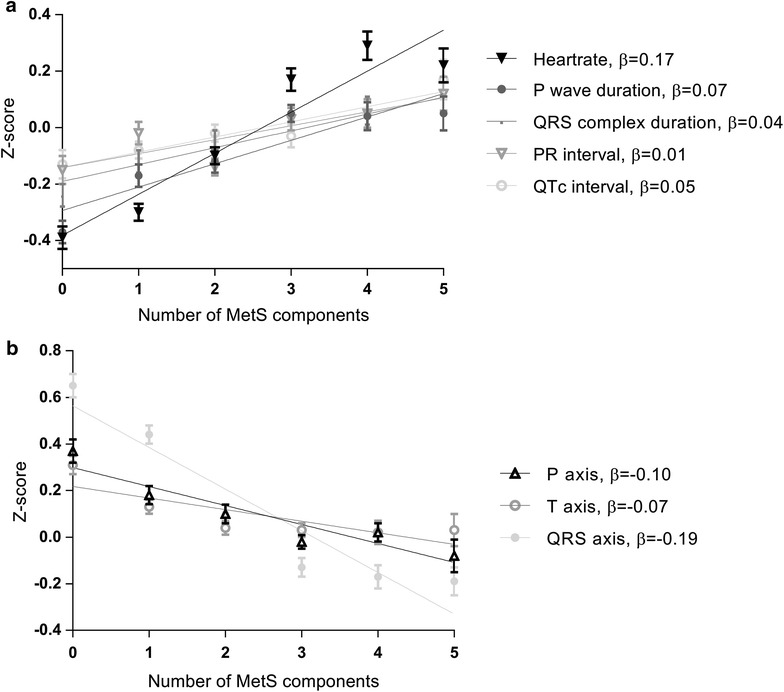
ECG parameters in relation to metabolic syndrome score. Regression lines are shown for the different ECG parameters. ECG parameters are expressed in Z-scores. **a** ECG parameters that increased with increasing number of metabolic syndrome components; **b** ECG parameters that decreased with increasing number of metabolic syndrome components. Results were based on weighted analysis adjusted for age, sex, ethnicity, smoking, alcohol intake, education level, physical activity and statin use

Figure 1 displays regression lines per SD of the ECG parameters used in a linear regression analysis per MetS component, with panel A showing all ECG parameters that increased and panel B showing all ECG parameters that decreased with increasing number of components. Per additional MetS component, heart rate, P wave duration, QRS complex duration and QTc interval increased and P-, T- and QRS axis decreased. In Table 3, the association of MetS score, ranging from zero to five, with Z-scores of ECG parameters is shown for obese and non-obese participants separately. For P wave duration (p = 0.027), P axis (p = 0.001), T axis (p < 0.001) and QRS axis (p < 0.001) the interaction terms between MetS score and obesity were statistically significant. In the non-obese participants, with each additional MetS component, P wave duration was 0.05 SD (95% CI 0.02, 0.09) longer, P axis was 0.08 SD (0.05, 0.12) lower, T axis was 0.07 SD (0.04, 0.11) lower and QRS axis was 0.17 SD (0.13, 0.21) lower. In the obese participants, P wave duration, P axis and T axis were not associated with increasing amount of MetS components, whereas QRS axis was 0.05 SD (0.02, 0.08) SD lower with each additional component, but this was less than in the non-obese participants. Furthermore, in non-obese participants percentage small Q-waves increased with each additional MetS component (4.5; 4.7; 6.8; 7.5; 11.6; 12.3%), while this was less clear in obese participants (0; 6.1; 8.8; 9.6; 9.2; 8.3%).

**Table 3:** Relation of standardized ECG parameters to metabolic syndrome scores (range 0–5)

ECG parameter	All	Non-obese (84%)	Obese (16%)	p value int
Heart rate	0.17 (0.15, 0.19)	0.16 (0.13, 0.19)	0.12 (0.09, 0.16)	0.137
P duration	0.07 (0.05, 0.10)	0.05 (0.02, 0.09)	0.00 (−0.04, 0.03)	0.027
QRS duration	0.04 (0.01, 0.06)	0.02 (−0.01, 0.06)	0.04 (0.00, 0.07)	0.466
PR interval	0.01 (−0.02, 0.04)	0.00 (−0.04, 0.04)	0.01 (−0.03, 0.04)	0.866
QTc interval	0.05 (0.02, 0.08)	0.04 (0.00, 0.07)	0.05 (0.01, 0.09)	0.335
P axis	−0.10 (−0.12, −0.07)	−0.08 (−0.12, −0.05)	−0.01 (−0.05, 0.02)	0.001
T axis	−0.07 (−0.10, −0.05)	−0.07 (−0.11, −0.04)	0.04 (0.00, 0.08)	<0.001
QRS axis	−0.19 (−0.22, −0.16)	−0.17 (−0.21, −0.13)	−0.05 (−0.08, −0.02)	<0.001

Data are presented as β (95% CI); change in Z-score of ECG parameter per metabolic syndrome component. Results were based on weighted analysis. Multivariate model adjusted for age, sex, ethnicity, smoking, alcohol intake, education level, physical activity and statin use

In a joint model including all separate MetS components together with potential confounding factors (Table 4), high waist circumference was associated with all ECG parameters. High waist circumference was associated with 2.4 bpm (95% CI 1.6, 3.2) higher heart rate, 3.4 ms (2.2, 4.4) longer P wave duration, 1.0 ms (0.1, 1.9) longer QRS complex duration, 2.4 ms (0.6, 4.3) longer PR interval, 2.7 ms (1.0, 4.3) longer corrected QT interval, 7.4° (5.3, 9.4) lower P axis, 5.2° (3.3, 7.1) lower T axis, 12.9° (10.5, 15.2) lower QRS axis and 1.9% (0.2, 3.6) more small Q-waves. In this joint model, hypertriglyceridemia was only associated with heart rate, reduced serum HDL-cholesterol with P wave duration, PR interval and QRS axis, increased blood pressure with heart rate, P wave duration, QRS complex duration, corrected QT interval, T axis and QRS axis and hyperglycemia with heart rate and PR interval.

**Table 4:** Influence of metabolic syndrome components on ECG parameters

ECG parameter	Waist circumference	Triglycerides	HDL-cholesterol	Blood pressure	Fasting glucose
Heart rate (bpm)	2.4 (1.6, 3.2)	3.2 (2.2, 4.1)	−0.3 (−1.2, 0.7)	1.8 (1.1, 2.5)	2.2 (1.4, 3.1)
P duration (ms)	3.4 (2.3, 4.4)	−0.6 (−1.9, 0.7)	1.7 (0.3, 3.0)	1.1 (0.0, 2.2)	−0.8 (−2.0, 0.4)
QRS duration (ms)	1.0 (0.1, 1.9)	−0.5 (−1.5, 0.6)	0.2 (−0.8, 1.2)	1.4 (0.4, 2.4)	−0.1 (−1.1, 1.0)
PR interval (ms)	2.4 (0.6, 4.3)	−1.5 (−3.9, 0.8)	3.6 (1.2, 6.1)	0.4 (−1.6, 2.5)	−3.5 (−5.4, −1.6)
QTc interval (ms)	2.7 (1.0, 4.3)	0.1 (−2.0, 2.1)	1.8 (−0.1, 3.8)	1.9 (0.2, 3.7)	−0.9 (−2.7, 1.0)
P axis (°)	−7.4 (−9.4, −5.3)	−0.6 (−2.9, 1.6)	−2.0 (−4.6, 0.6)	−1.9 (−4.1, 0.2)	1.0 (−1.3, 3.2)
T axis (°)	−5.2 (−7.1, −3.3)	−0.2 (−2.5, 2.2)	−2.0 (−4.4, 0.4)	−2.7 (−4.7, −0.8)	1.2 (−0.8, 3.2)
QRS axis (°)	−12.9 (−15.2, −10.5)	−2.5 (−5.5, 0.5)	−4.5 (−7.7, −1.4)	−5.6 (−8.2, −3.0)	−2.7 (−5.4, 0.0)
Small Q-wave (%)	1.9 (0.2, 3.6)	1.6 (−0.7, 3.9)	0.7 (−1.7, 3.2)	1.0 (−0.8, 2.8)	0.4 (−1.6, 2.3)

Data are presented as β (95% CI). Results were based on weighted analysis

Multivariate model adjusted for age, sex, ethnicity, smoking, alcohol intake, education level, physical activity, statin use and the other MetS components

## Discussion

We observed that electrocardiographic markers of subclinical CVD differed between participants with and without MetS, indicating more subclinical CVD in participants with than without MetS. ECG parameters associated with subclinical CVD increased with every additional MetS component present in an individual and for P wave duration, P axis, T axis and QRS axis, these associations were stronger in the non-obese than the obese population. Furthermore, in joint models including all MetS components high waist circumference was associated with all ECG parameters whereas results for the other components were less strong.

In this study, subclinical CVD was investigated by looking at subtle changes in ECG parameters. Although the observed changes are small and not per se of direct prognostic significance on the individual level, they give more insight on a population level. All ECG parameters investigated, have previously been associated with a broad range of future cardiovascular abnormalities, events or mortality [16–18, 23–27].

Differences between participants with and without MetS have also been demonstrated in other studies. In a study with 6765 participants aged 45–84 years, MetS was associated with ECG abnormalities [28]. The association of MetS with higher heart rate and also lower heart rate variability, indicative of an adverse effect of MetS on the cardiac autonomic modulation has previously been reported in literature [29, 30]. Furthermore MetS has been associated with borderline or abnormal T axis [31]. Furthermore, in a study that included both individuals with MetS as well as their offspring, evidence of early subclinical cardiovascular damage was found in individuals with MetS as well as their offspring [32]. In our study associations between MetS and ECG parameters are confirmed in a large group of extensively phenotyped individuals and on top of that we showed that it is important to pay attention to asymptomatic patients, with just zero or one component present, to prevent the increase of MetS components in these individuals and thereby also the development of CVD. We showed that this is already important in the non-obese population, since the associations of MetS with subclinical CVD are also present in this population.

We observed associations between the presence of MetS and also MetS score and ECG parameters. Thus far, literature is inconclusive about the risk associated with MetS components and increasing MetS score. In a study with 9406 participants, it was concluded that MetS was not associated with 1-year mortality, while reduced HDL-cholesterol was associated with higher risk and raised triglycerides were associated with lower 1-year mortality risk [5]. In a case–control study, MetS was associated with higher risk of venous thromboembolism. However, after multivariate analysis, only abdominal obesity was associated with higher risk [4]. There are also studies stating that increasing MetS score can be used as a risk factor for CVD [33, 34]. Moreover, it was shown that MetS is associated with increased heart failure risk in individuals without diabetes or baseline macrovascular complications [35].

Associations between ECG parameters and MetS score seemed stronger in the non-obese population. A possible explanation is that obese participants are more likely to have a high waist circumference and also worse ECG parameters, so they have less variation in their MetS score which varies from zero to five. Nevertheless, our study shows that in non-obese participants every additional component contributes to the association with subclinical CVD. However, pathophysiological mechanisms underlying this difference between non-obese and obese participants remain unclear from existing literature.

Furthermore, in the analysis of MetS components separately (Table 4), we are aware that when adjusting the associations of increased waist circumference and subclinical CVD, it is possible that adjustments are made for factors that might partially be in the causal pathway. However, this would dilute the associations, so the true association between increased waist circumference and subclinical CVD might even be stronger than the association that we find.

It is inconclusive through which mechanisms obesity or increased waist circumference lead to ECG abnormalities. Possibilities are increased sympathetic system activity, elevation of the diaphragm and increased cardiac output leading to left ventricular hypertrophy [36, 37]. Body fat was associated with measures of sympathetic activation in subjects with structurally normal hearts in the NEO study [38]. The leftward (superior) shifts of the T-, P- and QRS axis were associated with obesity in other studies [39–41]. Obesity can lead to an increase in cardiac loading and remodelling of the heart muscle leading to PR interval lengthening [42]. Also hormones produced by the adipose tissue influence the myocardial matrix, resulting in electrophysiological remodelling [42]. Also endovascular effects of obesity are present, induced by paracrine hormone expression of the adipose tissue that could alter the atrial function [43].

A strength of this study is the large study population (n = 6114) and the extensive measurements of potential confounding factors that were performed in the NEO study. A weakness is its observational cross-sectional design, precluding any conclusions about causality of the observed relationships.

On the basis of its observed relation with ECG parameters, we conclude that MetS is associated with subtle changes in ECG parameters, indicative of more subclinical CVD. As these ECG parameters are predictive of CVD and MetS score was associated with higher values of these parameters, MetS could be used as an early marker for subclinical CVD risk stratification, and to manage risks already in an early disease state. Furthermore, prevention of the development of MetS components is important in obese, but also very relevant in non-obese individuals.

## Conclusions

Metabolic syndrome score and its individual components, in particular abdominal obesity, are associated with ECG markers of subclinical CVD in both obese and non-obese persons.

## Additional file

**Additional file 1: Table S1.** Differences in baseline ECG parameters between participants with and without metabolic syndrome.

## Abbreviations

MetS: metabolic syndrome
CVD: cardiovascular disease
BMI: Body Mass Index
NEO: Netherlands Epidemiology of Obesity
LUMC: Leiden University Medical Center
SQUASH: Short Questionnaire to Assess Health-enhancing physical activity
MET-h/week: hours per week of metabolic equivalents
