# Supplementary material for: Association of metabolic syndrome and electrocardiographic markers of subclinical cardiovascular disease
Source: Diabetol Metab Syndr. 2017 May 22;9:40. doi: 10.1186/s13098-017-0238-9 (PMC5441065; doi:10.1186/s13098-017-0238-9)
Supplement: Supplementary file 1 — Additional file 1: Table S1. Differences in baseline ECG parameters between participants with and without metabolic syndrome. [file 13098_2017_238_MOESM1_ESM.docx]

| **Supplementary Table1 . Differences in baseline ECG parameters between participants with and without metabolic syndrome** | | | | |
| --- | --- | --- | --- | --- |
|  | Metabolic syndrome | |  | |
| ECG parameter | No (76%) | Yes (24%) | Diff (95%CI) | Diff (95%CI)^1^ |
| Heart rate (bpm) | 62.6 (0.2) | 68.1 (0.3) | 5.5 (4.7 , 6.2) | 5.2 (4.4 , 6.0) |
| P duration (ms) | 111.2 (0.3) | 114.9 (0.4) | 3.7 (2.8 , 4.7) | 2.8 (1.8 , 3.7) |
| QRS duration (ms) | 92.5 (0.3) | 94.6 (0.3) | 2.1 (1.3 , 3.0) | 1.2 (0.3 , 2.1) |
| PR interval (ms) | 162.2 (0.6) | 165.7 (0.6) | 3.5 (1.8 , 5.2) | 2.0 (0.2 , 3.8) |
| QTc interval (ms) | 413.9 (0.5) | 416.2 (0.6) | 2.3 (0.8 , 3.9) | 2.4 (0.9 , 4.0) |
| P axis (°) | 48.3 (0.6) | 42.7 (0.6) | -5.6 (-7.3 , -3.9) | -5.3 (-7.2 , -3.5) |
| T axis (°) | 39.0 (0.6) | 35.5 (0.7) | -3.5 (-5.3 , -1.7) | -3.1 (-5.0 , -1.1) |
| QRS axis (°) | 38.7 (0.8) | 21.1 (0.9) | -17.6 (-19.9 , -15.3) | -14.5 (-16.8 , -12.2) |
| Small Q-wave (%) | 5.3 | 9.2 | 3.8 (2.1 , 5.6) | 3.0 (1.1 , 4.8) |

Data are presented as mean (se) or percentage. Results were based on weighted linear regression analysis.

^1^Multivariate model adjusted for age, sex, ethnicity, smoking, alcohol intake, education level, physical activity and statin use
